# Nonlinear climatic sensitivity to greenhouse gases over past 4 glacial/interglacial cycles

**DOI:** 10.1038/s41598-017-04031-x

**Published:** 2017-07-04

**Authors:** Li Lo, Sheng-Pu Chang, Kuo-Yen Wei, Shih-Yu Lee, Tsong-Hua Ou, Yi-Chi Chen, Chih-Kai Chuang, Horng-Sheng Mii, George S. Burr, Min-Te Chen, Ying-Hung Tung, Meng-Chieh Tsai, David A. Hodell, Chuan-Chou Shen

**Affiliations:** 1 High-Precision Mass Spectrometry and Environment Change Laboratory (HISPEC), Department of Geosciences, National Taiwan University, Taipei, 10617 Taiwan, ROC; 2 Department of Earth Sciences, University of Cambridge, Cambridge, Cambridgeshire CB2 3EQ UK; 3 State Key Laboratory of Isotope Geochemistry, Guangzhou Institute of Geochemistry, Chinese Academy of Sciences, Guangzhou, 510640 People’s Republic of China; 4 Research Center for Environmental Changes, Academia Sinica, Taipei, 11529 Taiwan, ROC; 5 Institute of Applied Mechanics, National Taiwan University, Taipei, 10617 Taiwan, ROC; 6 Department of Earth Sciences, National Taiwan Normal University, Taipei, 11677 Taiwan, ROC; 7 NSF-Arizona Accelerator Mass Spectrometry Laboratory, University of Arizona, Tucson, AZ 85721 USA; 8 Institute of Applied Geosciences, National Taiwan Ocean University, Keelung, 20224 Taiwan, ROC

## Abstract

The paleoclimatic sensitivity to atmospheric greenhouse gases (GHGs) has recently been suggested to be nonlinear, however a GHG threshold value associated with deglaciation remains uncertain. Here, we combine a new sea surface temperature record spanning the last 360,000 years from the southern Western Pacific Warm Pool with records from five previous studies in the equatorial Pacific to document the nonlinear relationship between climatic sensitivity and GHG levels over the past four glacial/interglacial cycles. The sensitivity of the responses to GHG concentrations rises dramatically by a factor of 2–4 at atmospheric CO_2_ levels of >220 ppm. Our results suggest that the equatorial Pacific acts as a nonlinear amplifier that allows global climate to transition from deglacial to full interglacial conditions once atmospheric CO_2_ levels reach threshold levels.

## Introduction

Rapidly rising anthropogenic CO_2_ emissions over the past few decades pose a risk to human society and the biosphere. Relative to pre-industrial levels, the CO_2_ concentration is projected to double to 560 ppm from 2050–2100, increasing the global mean temperature by 2.0–4.5 °C by the end of the 21^st^ century. This warming is predicted to lead to large-scale ice sheet melting, shifts in rainfall patterns, and sea level rise^1^. Identifying the interactions among various forcing agents and feedback processes in the projected warming scenario is crucial to our understanding of social/biosystem sustainability and climate change mitigation strategies.

The tropical Pacific Ocean is the most important water vapor and moisture source to middle and high latitudes on the planet^2^. Accurately estimating tropical sea surface temperature (SST) responses to greenhouse gas (GHG)-induced radiative forcing (RF_GHG_) is fundamental to reliable predictions. Past climate studies^3, 4^, however, assumed that the sensitivity of tropical ocean SST to atmospheric GHG content was linear. Physical model simulations were limited by a lack of pre-industrial observational records for climate transitions from low to high GHG levels. The energy budget of the global climate system is, therefore, difficult to precisely evaluate. This severely hinders our understanding of the mechanisms and energy interactions among climatic sub-systems.

The sensitivity of tropical Pacific Ocean SST to RF_GHG_, mainly contributed by CO_2_ and <5% by CH_4_, during the past glacial/interglacial (G/IG) cycles was addressed^3–5^. Linear regression analyses^3, 4, 6^ were conducted to evaluate the SST response to changes in pCO_2_ assuming that the RF_GHG_ is the main driver of tropical SST and that the tropical Pacific SST would be 33–36 °C under a doubled CO_2_ scenario. By comparing the atmospheric pCO_2_ and SST values of the early Pleistocene to those of the late Pliocene, *Martinez*-*Boti et al*.^5^ found a lower sensitivity under high atmospheric pCO_2_ conditions, implying that the sensitivity may not respond linearly to the GHG level. Recent transient models^7^ based on composite paleo-temperature records also revealed nonlinear sensitivity patterns since 800 thousand years ago (kyr BP, before 1950 AD). These variable responses to RF_GHG_ are supported by a conceptual and dynamic-flow box modeling study^8^ that argues for the existence of possible step-wise and/or different equilibrium climate states during G/IG cycles.

This dispute, linear versus nonlinear climatic sensitivities, hinders our understanding of climate evolution and our ability to evaluate the fidelity of global energy simulations under the current warming stress. Orbital-scale high-resolution SST records are required to clarify this debate. Here, we combine a new record of southern Western Pacific Warm Pool (WPWP) SST anomalies (ΔSST) and previously published equatorial Pacific records^4, 9–11^ for the past four G/IG cycles to document the non-linearity of the relationship between equatorial Pacific SSTs and GHG levels and to identify the specific pCO_2_ threshold.

**Figure 1:**
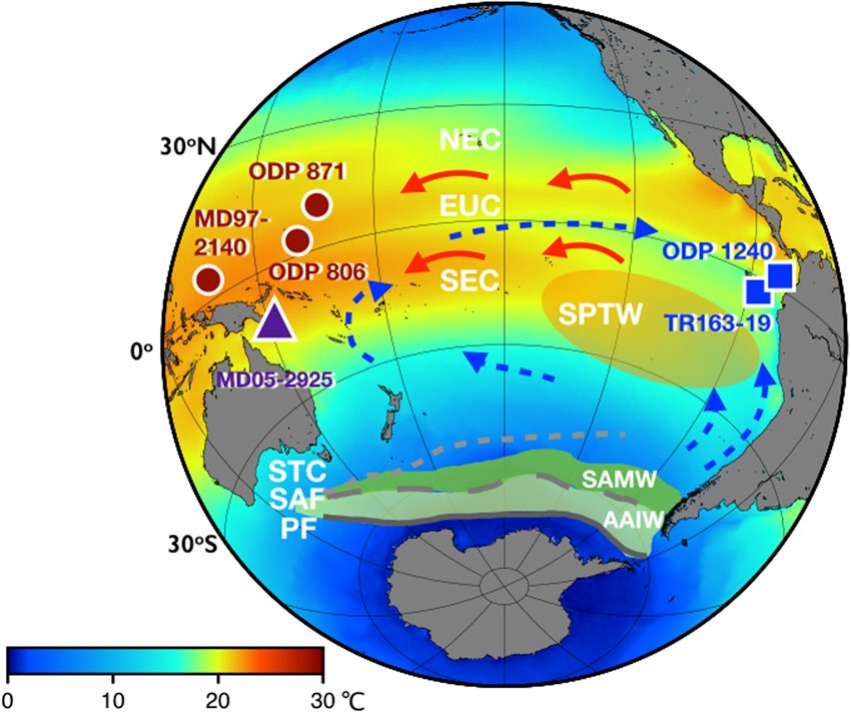
A sea surface temperature map with site locations, schematic circulation, and distribution of water masses. The purple triangle represents site MD05-2925. Dark red circles and blue squares are the selected sites in the equatorial Pacific. Gray solid, long dashed, and short dashed lines show the Polar Front (PF), Subantarctic Front (SAF), and Subtropical convergence zone (STC), respectively. Gray and green shadings denote the formation regions of Antarctic Intermediate Water (AAIW) and Subantarctic Mode Water (SAMW), respectively. Light orange shading represents the South Pacific Tropical Water (SPTW). Blue dashed arrays represent the undercurrent pathways of the Equatorial Under Current (EUC and water masses from southern ocean). Red solid arrows represent the surface South Equatorial Current (SEC) and North Equatorial Current (NEC). The AAIW and SAMW (gray and green shadings) flow from the southern hemisphere high latitudes to the SPTW region and spread out to the South Pacific Ocean through the EUC and EEP wind-driven upwelling system and resurfacing processes through water mass mixing on the scale of decades^20^. This map was generated with Generic Mapping Tools (GMT) version 5 (ref. 40). Global satellite mean annual sea surface temperatures during 2009–2013 with color coding are from the National Aeronautics and Space Administration (NASA) Ocean Color database (http://oceancolor.gsfc.nasa.gov)^41^.

A marine sediment core, MD05-2925 (9.3°S, 151.5°E, water depth 1661 m), was retrieved from the Solomon Sea east of Papua New Guinea (Figs 1, S1). The age model for MD05-2925 was established using radiocarbon dates^12^ and composite benthic foraminiferal oxygen isotopic stratigraphy (Figs S2, S3). The Mg/Ca ratios of planktonic foraminifera, *Globigerinoides ruber*, were determined using a sector field inductively coupled plasma mass spectrometer (SF-ICP-MS)^13^ and were used to establish a 360-kyr SST sequence with 200- to 900-year resolution.

## Results

### Foraminiferal Mg/Ca-inferred Solomon Sea SST

**Figure 2:**
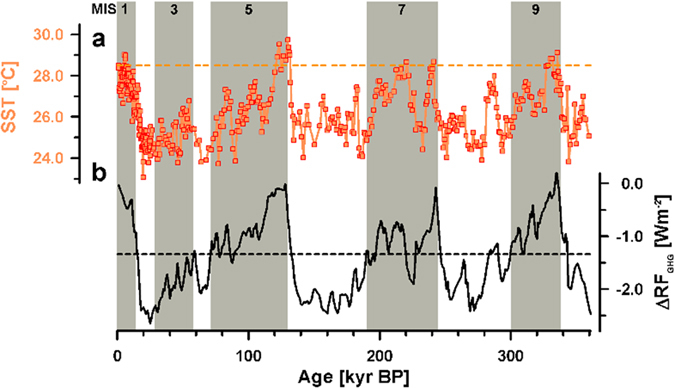
MD05-2925 SST values and ΔRF_GHG_ of greenhouse gases. (**a**) Mg/Ca-inferred SST in the Solomon Sea. (**b**) Calculated ΔRF_GHG_ (see Methods). Gray bars indicate interglacial periods. The orange and black dashed lines are the modern annual average SST (28.5 °C) and the CO_2_ = 220 ppm, respectively.

The new reconstructed Solomon Sea SST record shows a large G/IG change of approximately 4 °C, which is supported by a previous study^14^ (Fig. 2a). The interglacial temperatures of 28–29 °C during Marine Isotope Stage (MIS) 7 and MIS 9 are similar to those of the Holocene and are higher by 1–2 °C than those of MIS 5. Large SST variations of 2–3 °C can be observed during glacial periods, such as MIS 2–4, 6 and 8.

Spectral analysis (Fig. S5) shows that the Solomon Sea SST record is characterized by strong obliquity pacing and a good correlation with Antarctic temperature changes during the past four G/IG cycles. The Southern Hemisphere (SH) middle- to high-latitude climate apparently exerts a significant influence on the Solomon Sea, possibly by oceanic tunnel connections^11, 12^ (Figs S5–S9).

The statistical correlation shown in Figure S7 suggests that the SST changes in the Solomon Sea could be related to SH high-latitude climate variation, similar to the eastern equatorial Pacific (EEP) upwelling region^11^. The trends in the time series of Antarctic temperature and southern WPWP SST have been similar since 360 ka (R^2^ = 0.59 or 0.65 if MIS 7 is omitted, Fig. S7). This correlation implies a persistent teleconnection between the two regions (Figs S7–S9)^12^. Therefore, the southern WPWP and EEP regions are linked to Southern Ocean climate and/or affected directly by the RF_GHG_^3, 15, 16^.

### Climate sensitivity and regional comparisons

**Figure 3:**
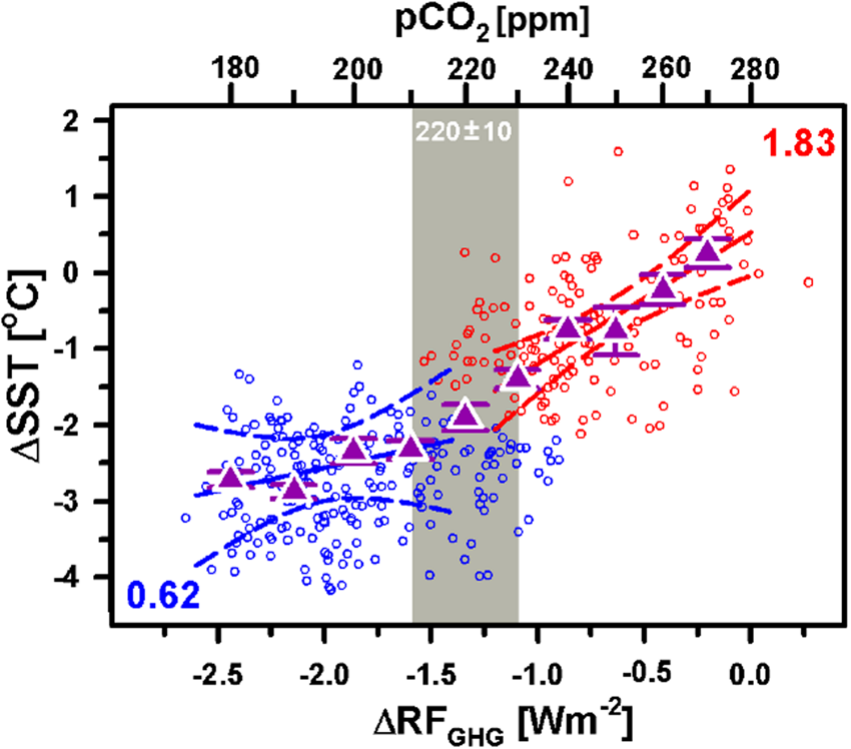
Nonlinearity of SST sensitivity to climate in the southern WPWP. The Solomon ΔSST and greenhouse gas radiative forcing ΔRF_GHG_ data are plotted at a 1-kyr interval. Two groups (blue and red circles) were divided at a pCO_2_ level of 220 ppm via cluster analysis (see Methods). Purple triangles are standard deviations of the mean for ΔSST data points at a segment of radiative forcing corresponding to 10 ppm pCO_2_. Solid and dashed lines for each group represent the regression line and 95% confidence interval, respectively. The determined slopes are given as lines. The gray vertical bar marks the significant difference threshold for the non-linear SST changes at a pCO_2_ level of 220 ± 10 ppm.

The relationship between tropical ΔSST and the atmospheric RF_GHG_ anomaly (relative to pre-industrial levels, ΔRF_GHG_) for the Solomon Sea record is plotted in Figure 3. This comparison expresses a striking nonlinear correlation with a significant change at an atmospheric CO_2_ threshold of 220 ± 10 ppm (Fig. 3). Southern WPWP ΔSST values increase from −4.1 to −1.2 °C as the ΔRF_GHG_ values increase from −2.75 to −1.55 W m^−2^, corresponding to a pCO_2_ range of 170–210 ppm. Thus, the sensitivity is 0.62 ± 0.33 °C (W m^−2^)^−1^. Note that all the sensitivity uncertainties are given in 1σ range (Table S3). During the second interval, ΔRF_GHG_ increases from −1.55 to 0 W m^−2^ (210–280 ppm pCO_2_). The sensitivity increases to 1.83 ± 0.17 °C (W m^−2^)^−1^ as ΔSST increases from −2.1 to 1.8 °C. No significant differences are observed in the SST-RF_GHG_ response among the past four G/IG periods at 1–70, 71–160, 161–270, and 271–360 kyr BP (Fig. S8), indicating that the correlation is independent of the G/IG cycles in this region.

**Figure 4:**
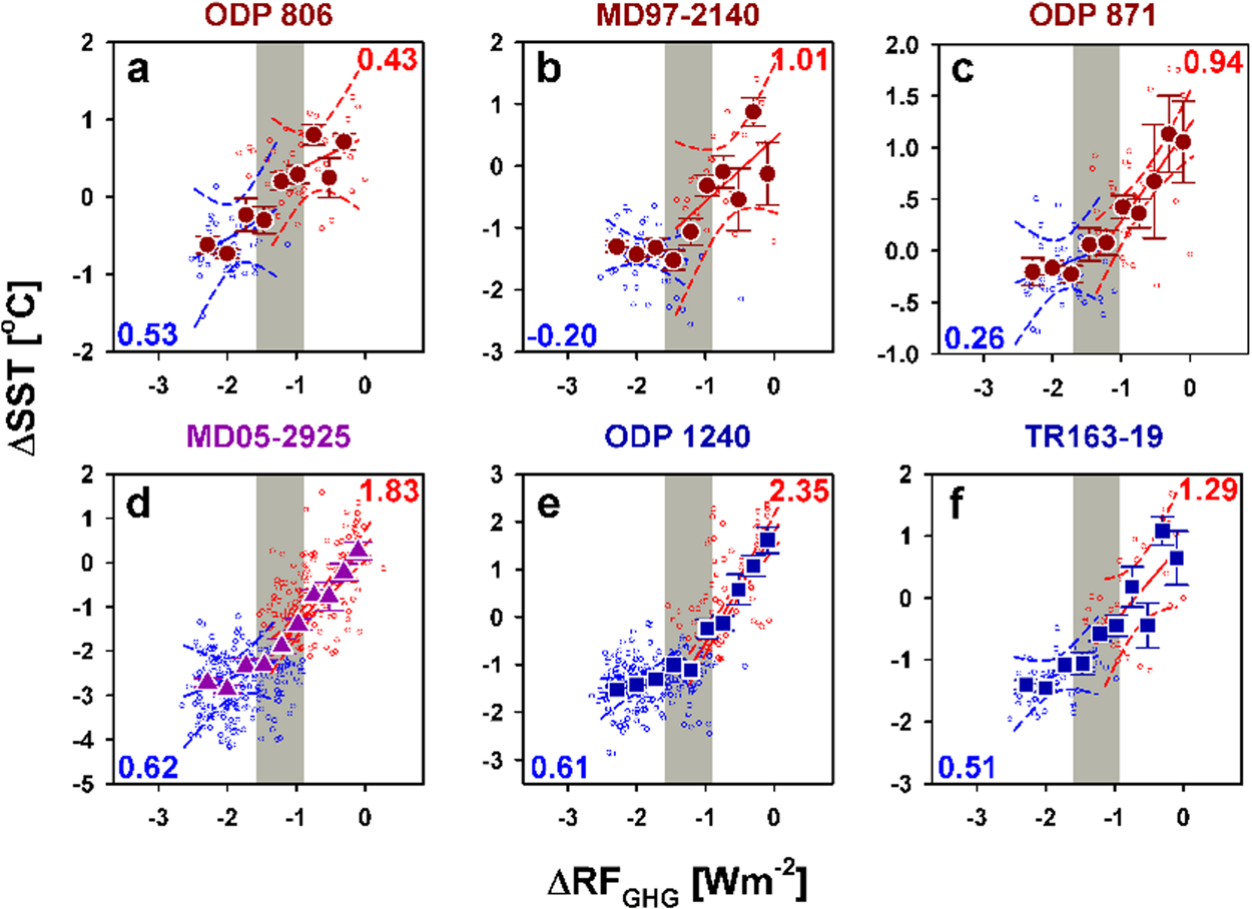
Comparison of tropical Pacific SST sensitivities. (**a–c**) Western Pacific with three sites: ODP 806, MD97-2140, and ODP 871 (refs 4, 9, 10). (**d**) Solomon Sea (site MD05-2925, this study). (**e**,**f**) Eastern equatorial Pacific with two sites: ODP 1240 and TR163-19 (refs 9, 11). Symbols, lines, slopes, and gray vertical bars are the same as in Figure 3 but for multiple sites. The data from three G/IG cycles are used in (**e**), and data from four G/IG cycles are used in the other panels.

A comparison of ΔSST and pCO_2_ values over the past 3–4 G/IG cycles at six western and eastern equatorial Pacific sites (Fig. 1) is shown in Figure 4. Five (MD05-2925, ODP 1240, TR163-19, MD97-2140, and ODP 871) of the six sites are characterized by a nonlinear SST-RF_GHG_ relationship with an atmospheric CO_2_ threshold of 220 ± 10 ppm (Fig. 4). Comparable to the southern WPWP site MD05-2925, the SH-affected EEP upwelling sites of ODP 1240 and TR163-19 also feature similar nonlinearity. Sites ODP 1240 and TR163-19 have low sensitivities of 0.61 ± 0.14 and 0.51 ± 0.14 °C (W m^−2^)^−1^, respectively, at pCO_2_ values of <220 ± 10 ppm and high sensitivities of 2.35 ± 0.17 and 1.29 ± 0.50 °C (W m^−2^)^−1^, respectively, at pCO_2_ values of >220 ± 10 ppm. A similar nonlinearity was also reported in the southern WPWP region over the past 400 kyr BP (Fig. 5 of ref. 15).

MD97-2140, ODP 871, and ODP 806 in the northern WPWP show more complicated patterns. MD97-2140 and ODP 871 have nonlinear relationships and sensitivities of −0.20 ± 0.15 to 1.01 ± 0.57 °C (W m^−2^)^−1^, respectively, which are less than the values of the EEP sites and MD05-2925. ODP 806 is the only site characterized by a linear trend due to an absence of statistics-based different sensitivities (Table S3). Its sensitivity is 0.70 ± 0.10 °C (W m^−2^)^−1^ (Fig. 4). The nonlinearity observed in the northern WPWP can be attributed to equatorial upwelling, regional oceanic dynamics, and topographic settings^4^, which might transport high latitude climatic effects to equatorial Pacific^17, 18^.

## Discussion

We hypothesize that this general nonlinear SST response is associated with the sea ice coverage in the Southern Ocean, the related release of ocean-derived CO_2_, and induced atmospheric radiative forcing during the last four G/IG cycles. *Jaccard et al*.^19^ presented sedimentary evidence that sea ice in the Southern Ocean could affect oceanic-atmospheric CO_2_ exchange by controlling the Subarctic Mode Water (SAMW) and Antarctic Intermediate Water (AAIW) formations during the past one million years. The SH high-latitude impact on the equatorial Pacific hydroclimate and the teleconnection between SAMW/AAIW and South Pacific Tropical Water (SPTW, Fig. 1) have been reported based on evidence from the subtropical gyre and resurfacing processes, such as upwelling and mixing^20^. The oceanic tunneling process represents the link between the Southern Ocean modulation and the WPWP and EEP. The possible processes are as follows. The gradual melting of sea ice in the Subantarctic Zone and the release of CO_2_ during the initial phase of glacial termination could cause the first stage of the SST-RF_GHG_ response. Positive latent and sensible heating likely then triggers a sudden retreat of Southern Ocean sea ice and a rapid increase in pCO_2_, leading to warming and a better ventilated SAMW/AAIW^21^. A pCO_2_ level of 220 ± 10 ppm can be considered the critical threshold for runaway melting and is supported by an Earth system model simulation^22^.

Our hypotheses are supported by previous climate reconstructions^23, 24^. Taking the last termination as an example, a pCO_2_ level of 220 ± 10 ppm occurred during Heinrich event 1, when meridional circulation shut down, the Southern Ocean warmed, and releases from the major deep-sea carbon reservoir began^23^. Benthic foraminiferal δ^13^C and SST data from the southwestern Pacific also suggest that the SAMW/AAIW experienced periods of relatively strong and weak ventilation^24^.

The responses of the WPWP and EEP thermal conditions to GHGs imply that the Southern Ocean–tropical ocean link acts as a nonlinear amplifier during glacial terminations via ocean circulation and atmospheric energy feedbacks. Once the pCO_2_ level exceeded 220 ppm, the southern WPWP SSTs increased by 1.83 °C (W m^−2^)^−1^, a rate that is three times higher than that associated with pCO_2_ levels of <220 ppm. The higher SST and increased latent heating may also have acted as a positive feedback mechanism, contributing to the collapse of high-latitude ice sheets^25^. Rapid warming in the equatorial Pacific possibly further weakened Hadley circulation^12^ and strengthened the SH westerlies. The enhanced Southern Ocean upwelling was then able to release more CO_2_ into the atmosphere^23^. Similar climatic threshold behavior during the past one million years of G/IG oscillations has been reported^19^. The teleconnection between the southern and northern hemisphere climates via WPWP and EEP SSTs during past G/IG cycles suggests that the tropical climate system plays a crucial role in rapid climatic transitions on Earth.

The clear relationship between equatorial Pacific SSTs and RF_GHG_ provides an important reference for understanding past and future climate dynamics. First, the mechanism that controls the nonlinearity in different oceans needs to be confirmed to improve predictions of the responses to increases in pCO_2_. Second, the potential existence of another higher CO_2_ threshold should be determined. *Martinez*-*Boti et al*.^5^ reported that the climate sensitivity could be similar or greater at higher pCO_2_ levels. The spatial variability in climate sensitivity during the Pliocene, with pCO_2_ levels of 300–500 ppm, remains unknown. More spatial and temporal high-resolution SST records and accurate GHG concentration reconstructions are required to clarify the complexity of the tropical ocean thermal response to RF_GHG_ changes and potential high-latitude teleconnections and to understand the linkages between tropical and high-latitude climate systems at different timescales.

## Methods

### Site MD05-2925

A marine sediment core was obtained at site MD05-2925 during the 2005 PECTEN (Past Equatorial Climate: Tracking El Niño) cruise, which was supported by the International Marine and Climate Changes (IMAGES) Project, on the research vessel Marion Dufresne. This site is located on the Woodlark rise, Solomon Sea, which is close to Papua New Guinea (PNG). The main route of the South Equatorial Current (SEC) traverses the Solomon Sea. The SEC originates in the mid-latitudes and travels to the equator. The annual average SST is 28.5 °C (ref. 26) and the seasonal variations are from 27.4 to 29.4 °C from the closest observational site at 8°S (ref. 27).

### Age model

The MD05-2925 age model was established using accelerator mass spectrometry radiocarbon (AMS ^14^C) dates and benthic foraminiferal oxygen isotope stratigraphic correlation. The compiled benthic foraminiferal δ^18^O data were correlated to the global benthic foraminiferal δ^18^O stack (LR04, ref. 28) (Figs S2, S3). All benthic foraminiferal tests were >250 μm in size, and 2–5 tests were used to measure δ^18^O values. Our group has reported the first 282-kyr age model^12, 29^ and adopt the interspecies δ^18^O offset constants from previous studies^29^. All oxygen isotope age control points are summarized in Table S1.

### Planktonic foraminiferal δ^18^O and Mg/Ca ratio

In total, 40–60 planktonic foraminifera *G*. *ruber* (white, *s*.*s*., 250–300 μm) were picked for oxygen isotopic measurements and Mg/Ca determinations. Each of the paired δ^18^O-Mg/Ca measurements, 20–30 tests were crashed and mixed for clearing procedure. For the oxygen isotope measurements, the samples were ultrasonicated 3–4 times, immersed in NaOCl for 24 hours, and dried. The cleaned samples were measured using an isotope ratio mass spectrometer (IRMS) at the National Taiwan Normal University. Analytical reproducibility is ±0.60‰ (2 RSD, N = 701)^12^ with respect to the Vienna Pee Dee Belemnite (VPDB). For the Mg/Ca measurements, the shells were gently crushed and placed in Teflon vials. The cleaning procedure followed that of *Lo et al*.^13^. Cleaned samples were dissolved in 5% HNO_3_ and measured using a Thermo-Finngan Element II sector field inductive coupled plasma mass spectrometer (SF-ICP-MS) at the High-Precision Mass Spectrometry and Environment Change Laboratory (HISPEC), Department of Geosciences, National Taiwan University. The external uncertainty is ± 0.60% (2 RSD). *G*. *ruber* δ^18^O and Mg/Ca values during the last termination (23-10 kyr BP) were previously reported^12^.

### SSTs inferred from *G*. *ruber* Mg/Ca ratios

The average Solomon Sea *G*. *ruber* Mg/Ca ratios vary from approximate 3.5 mmol mol^−1^ during the glacial periods to 4.5–5.0 mmol mol^−1^ during the interglacial periods (Fig. S4b). We adapted composite equation Mg/Ca = 0.38 x e^(0.09 x SST)^ to calculate the corresponding SST values^30, 31^. The average *G*. *ruber* shell weight during glacial periods (11.37 ± 1.11 μg, 1RSD, n = 20) does not differ from that during interglacial periods (11.46 ± 0.78 μg, 1RSD, n = 21) (Fig. S4d). The coarse fraction weight percentage shows no clear G/IG changes, which is reported to be linked with the carbonate preservation condition in the eastern equatorial Pacific region^32^ (Fig. S4c). Accordingly, no additional dissolution correction needs to be applied to the MD05-2925 Mg/Ca ratios, similar to the SST record reported in the northern PNG region^15^.

### ΔRF_GHG_ calculation

We reconstructed ΔSST (Eq. 1) and ΔRF_GHG_ (Eq. 2) by referencing the measured SSTs from the marine sediment core and the content of EPICA ice core greenhouse gases to the modern value.

1 $${\rm{\Delta }}\mathrm{SST}={{\rm{SST}}}_{{\rm{i}}}-{{\rm{SST}}}_{0}$$

ΔSST is defined as the difference between past temperatures (SST_i_) and the modern annual average temperature (SST_0_, 28.5 °C)^26^. ΔRF_GHG_ is defined as the difference between a certain past GHG level ([CO_2_] and [CH_4_]) and the pre-industrial greenhouse gas level ([CO_2_]_0_ = 280 ppm, [CH_4_]_0_ = 700 ppb)^33^. Although CH_4_ contributes only <5%, we calculated the ΔRF_GHG_ using both CO_2_ and CH_4_. The negative feedback from N_2_O is negligible with respect to RF^26^. The equation used to determine ΔRF_GHG_ is as follows:

2 $$\begin{array}{c}{{\rm{\Delta }}\mathrm{RF}}_{{\rm{GHG}}}={{\rm{\Delta }}\mathrm{RF}}_{{\rm{CO}}2}+{{\rm{\Delta }}\mathrm{RF}}_{{\rm{CH}}4}=4.841\,{\mathrm{ln}([\mathrm{CO}}_{2}]/{[{{\rm{CO}}}_{2}]}_{0})+0.0906{(\surd [{{\rm{CO}}}_{2}]\mbox{--}\surd [{{\rm{CO}}}_{2}])}_{0}\\ +0.036\,\mathrm{ln}(\surd [{{\rm{CH}}}_{4}])-(\surd {[{{\rm{CH}}}_{4}]}_{0}).\end{array}$$

### Regional sensitivity comparison

We summarized the updated *G*. *ruber* Mg/Ca records, which included at least three G/IG cycles, and calculated the relationships between ΔSST and ΔRF_GHG_. The records of ODP 806 (0.3°N, 159.4°E, water depth 2520 m)^9^, MD97-2140 (2.0°N, 141.7°E, water depth 2547 m)^10^, ODP 871 (5.6°N, 172.3°E, water depth 1255 m)^4^, TR163-19 (2.3°N, 91°W, water depth 2348 m)^9^, and ODP 1240 (0.0°N, 86.5°E, water depth 2921 m)^11^ were used. The Mg/Ca records of ODP 806 and TR163-19 have been recently calibrated using the dissolution correction equation^4^. We resampled ODP 806, ODP 871, TR163-19, and MD97-2140 at a 4-kyr interval and ODP 1240 at a 1-kyr interval and compared them to the contemporaneous resampled Antarctica ΔT and pCO_2_ records with the AICC2012 age model^34, 35^, following the methods of previous studies^4, 9^. No significant lead-lag difference is observed between the Antarctic and Solomon Sea ΔSST records (Table S2).

### Statistical methods to identify nonlinearity

#### Non-overlapping binned method

A non-overlapping binned method, used by the compilation of tropical SSTs (ref. 12), was applied to calculate the average trend of ΔSST versus ΔRF_GHG_. A radiative forcing window corresponding to 10 ppm pCO_2_ was used to calculate the average value of ΔSST and standard deviation of the mean for each of the SST data locations with different time resolutions.

#### Cluster analysis

K-means clustering analysis^36^ is widely used in diverse fields^37–39^ to statistically determine inflection points from separated k groups of any dataset with a non-linear trend. The data shown in all panels of Figures 3 and 4 were divided into two groups by k-means analysis. The regression lines with a 95% confidence interval of the two groups were calculated.

## Electronic supplementary material

Supplementary Information
