# Supplementary material for: Nonlinear climatic sensitivity to greenhouse gases over past 4 glacial/interglacial cycles
Source: Sci Rep. 2017 Jul 4;7:4626. doi: 10.1038/s41598-017-04031-x (PMC5496849; doi:10.1038/s41598-017-04031-x)
Supplement: Supplementary file 1 — Supplementary Information [file 41598_2017_4031_MOESM1_ESM.pdf]

SUPPLEMENTARY INFORMATION

Nonlinear climatic sensitivity to greenhouse gases over past 4 glacial/interglacial cycles

Li Lo[1,2,3]*, Sheng-Pu Chang[1], Kuo-Yen Wei[1], Shih-Yu Lee[4,]*, Tsong-Hua Ou[5], Yi-Chi Chen[1], Chih-Kai Chuang[1], Horng-Sheng Mii[6], George S. Burr[7,#], Min-Te Chen[8], Ying-Hung Tung[1], Meng-Chieh Tsai[1], David A. Hodell[2], and Chuan-Chou Shen[1,]*

1. High-Precision Mass Spectrometry and Environment Change Laboratory (HISPEC), Department of Geosciences, National Taiwan University, Taipei 10617, Taiwan ROC

2. Department of Earth Sciences, University of Cambridge, Cambridge, Cambridgeshire CB2 3EQ, United Kingdom

3. State Key Laboratory of Isotope Geochemistry, Guangzhou Institute of Geochemistry, Chinese Academy of Sciences, Guangzhou 510640, PRC

4. Research Center for Environmental Changes, Academia Sinica, Taipei 11529, Taiwan ROC

5. Institute of Applied Mechanics, National Taiwan University, Taipei 10617, Taiwan ROC

6. Department of Earth Sciences, National Taiwan Normal University, Taipei 11677, Taiwan ROC

7. NSF-Arizona Accelerator Mass Spectrometry Laboratory, University of Arizona, Tucson, AZ 85721, USA

8. Institute of Applied Geosciences, National Taiwan Ocean University, Keelung 20224, Taiwan ROC

*To whom correspondence should be addressed:

Chuan-Chou Shen: Tel: 886-2-33665878; Fax: 886-2-33651917; E-mail: river@ntu.edu.tw

Li Lo: Tel: 86-20-85290281; Fax: 86-20-85290130; E-mail: lilo@gig.ac.cn

Shih-Yu Lee: Tel: 886-2-27871925; Fax: 886-2-27871924; E-mail: shihyu@gate.sinica.edu.tw

[#]retired

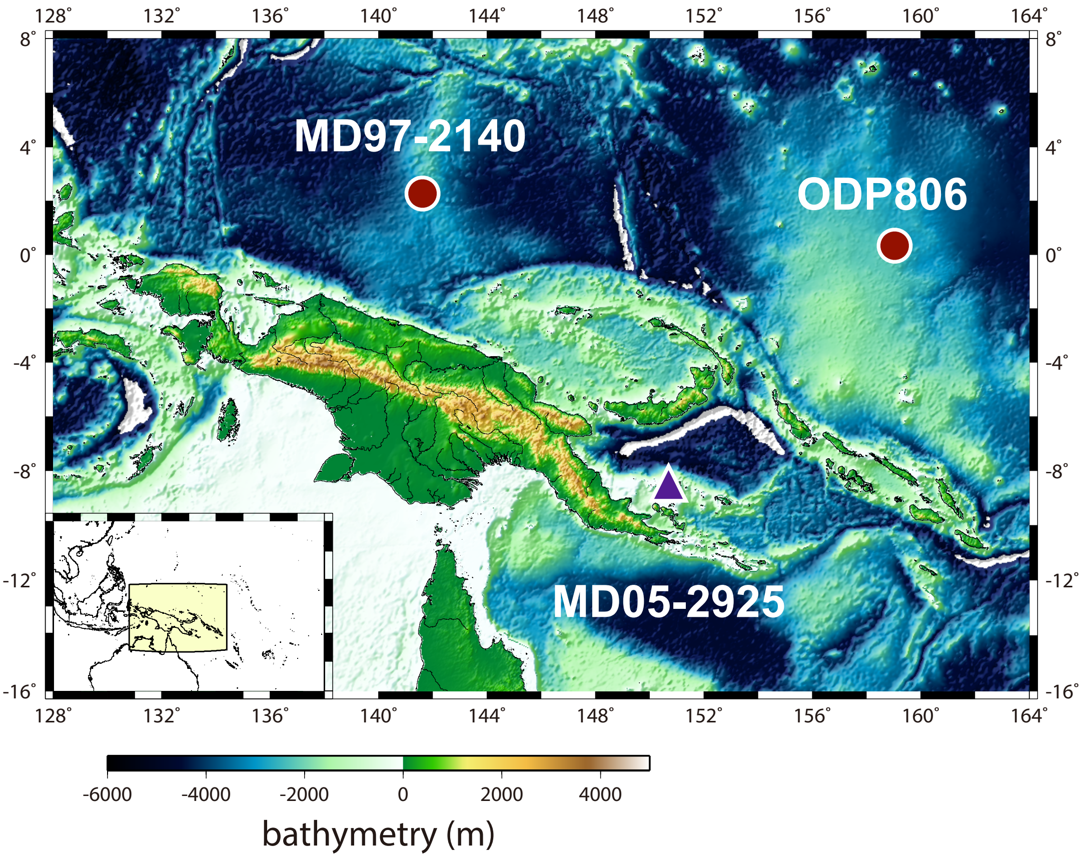

Supplementary Figure S1. Detail bathymetry map of Solomon Sea in the Papua New Guinea. This map was generated with Generic Mapping Tools (GMT) version 5 (ref. 1).

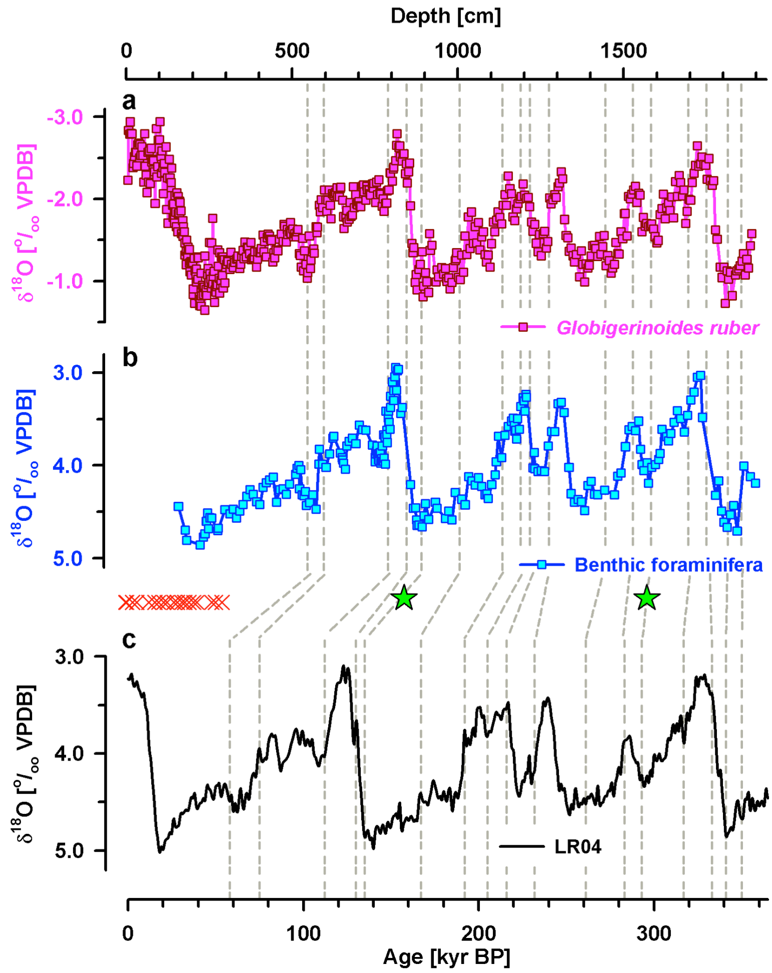

Supplementary Figure S2. Age model of core MD05-2925. (**a**) MD05-2925 *Globigerinoides ruber* $\delta^{18}O$ record. (**b**) A composite MD05-2925 benthic foraminiferal $\delta^{18}O$ record. Red cross symbols denote calibrated AMS ^{14}C dates used for upper 292 cm (ref. 2) and green stars are the two biostratigraphic events, which are the last occurrences of *G. ruber* (pink)[3] and first occurrence of *Emiliania huxleyi* at 833 and 1550 cm, respectively. Dashed lines are the age control points by comparing with (**c**) global composite stack LR04 (ref. 4).

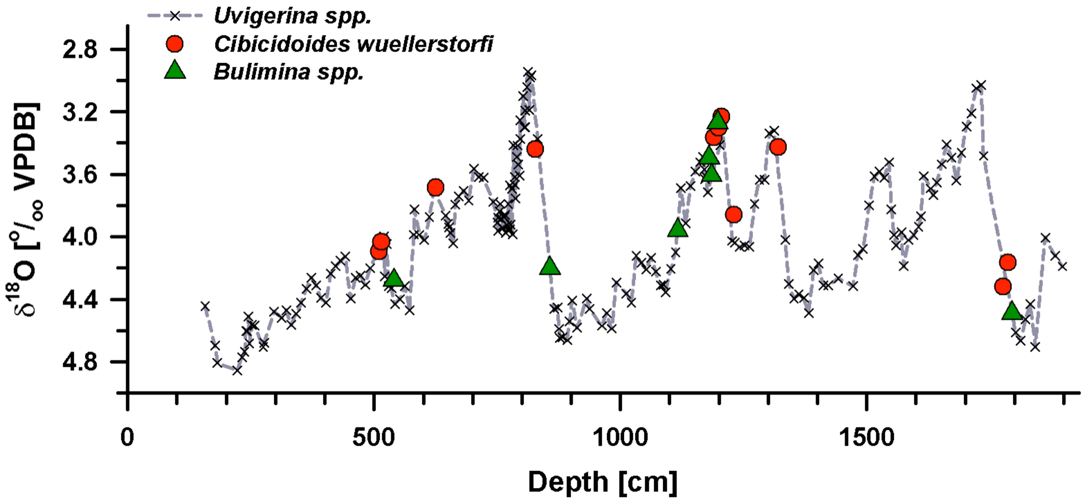

Supplementary Figure S3. A composite benthic foraminiferal oxygen isotope record of core MD05-2925. Dark gray dashed line with cross symbols denotes the oxygen isotope data for *Uvigerina spp.* Red circles and green triangles are the corrected $\delta^{18}O$ data of *Cibicidoides wuellerstorfi*[5] and *Bulimina spp.*[6], respectively.

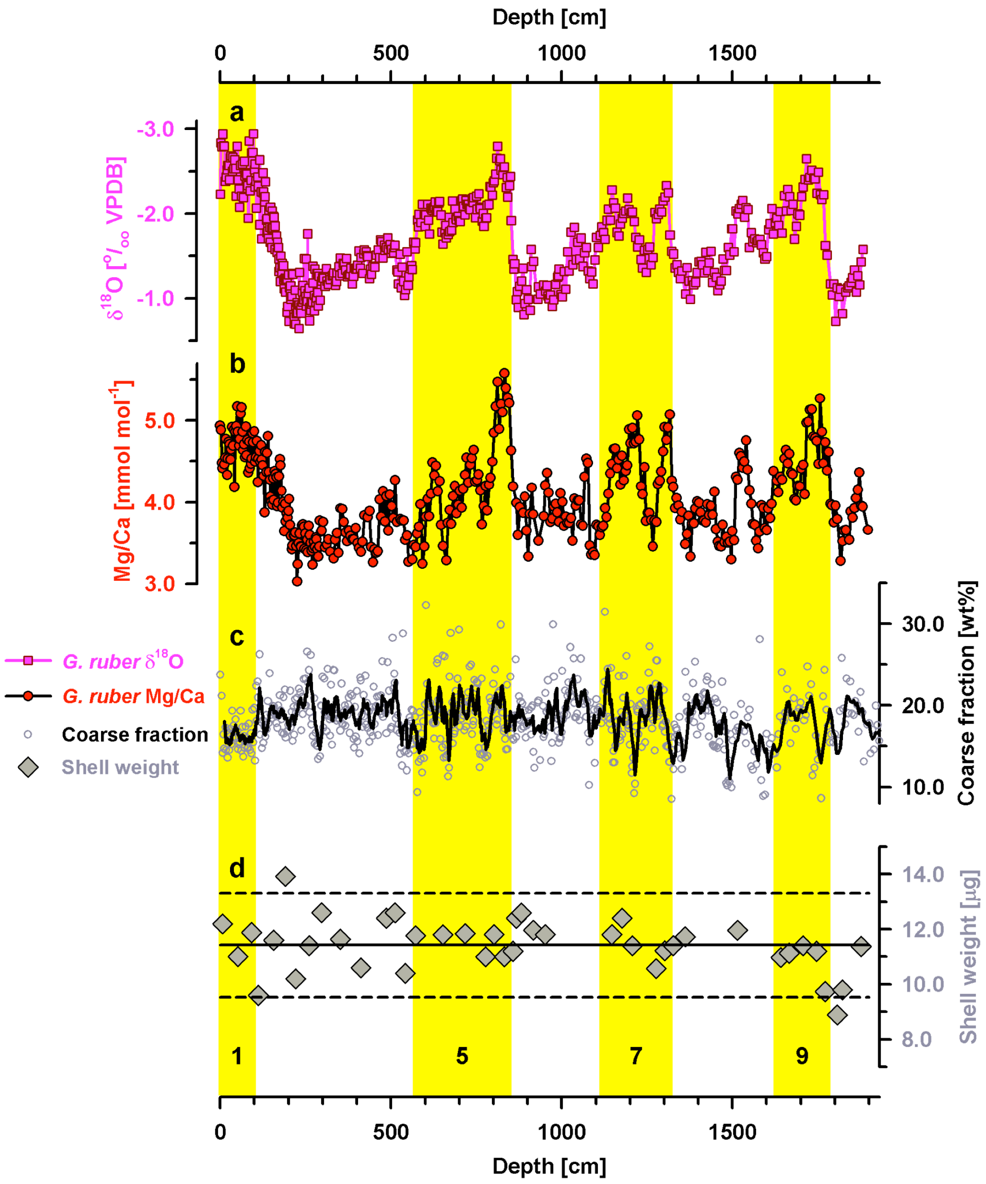

Supplementary Figure S4. MD05-2925 *G. ruber* Mg/Ca ratios. (**a**) *G. ruber* $\delta^{18}O$ (**b**) *G. ruber* Mg/Ca (**c**) coarse fraction (>63 **μ**m, weight percentage). The gray hollow dots are measured data points and the solid black line is 5-points running average (**d**) *G. ruber* shell weight (μg). The diamond dots are the *G. ruber* shell weight from 40-60 foraminiferal tests. The solid and dashed black lines are the average of shell weight during the study period and 2σ range, respectively (11.42 ± 1.90 μg).

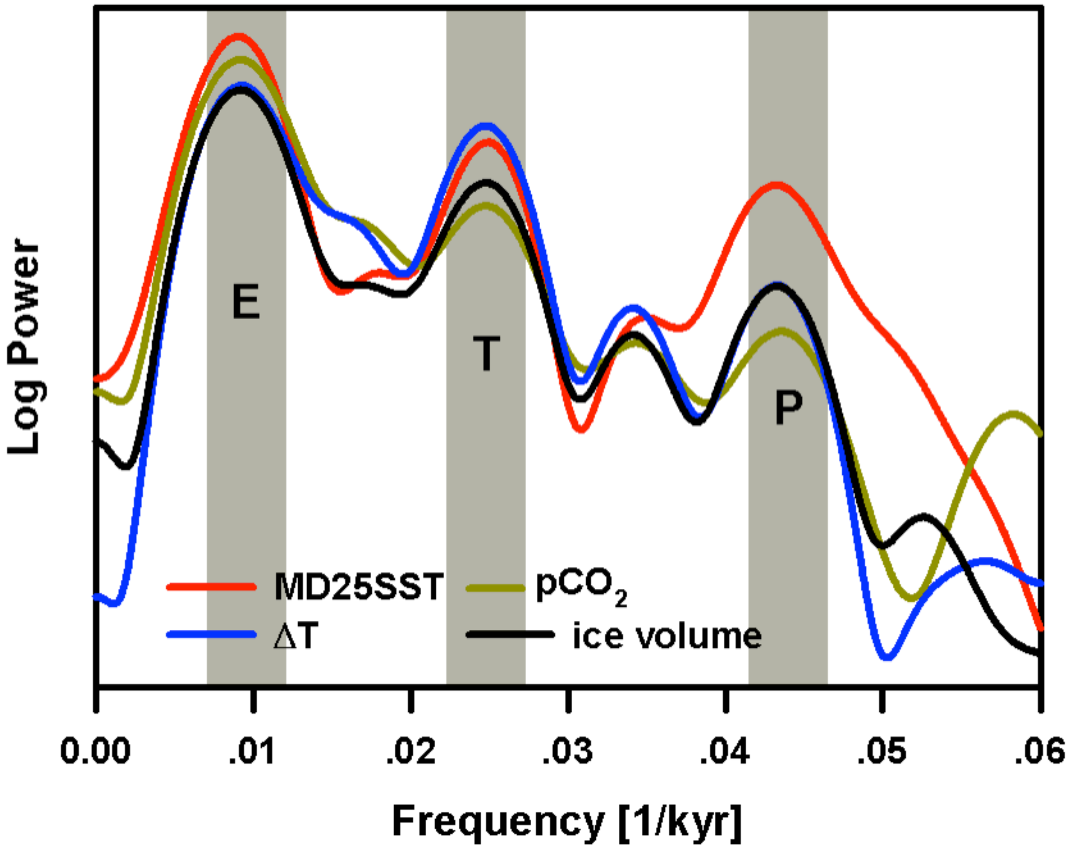

Supplementary Figure S5. Spectral analyses of paleo-proxy records. Records include MD05-2925 SST (red), LR04 composite represent global ice volume (black)[4], Antarctic temperature (blue)[7], and atmosphere CO_2 (yellow)[8] over the past 360 thousand years (kyr BP). All records were resampled into 1-kyr resolution and analyzed by Arand software[9]. Gray bars with letters E, T, and P, represent eccentricity, tilt (obliquity), and precession periodicity intervals, respectively.

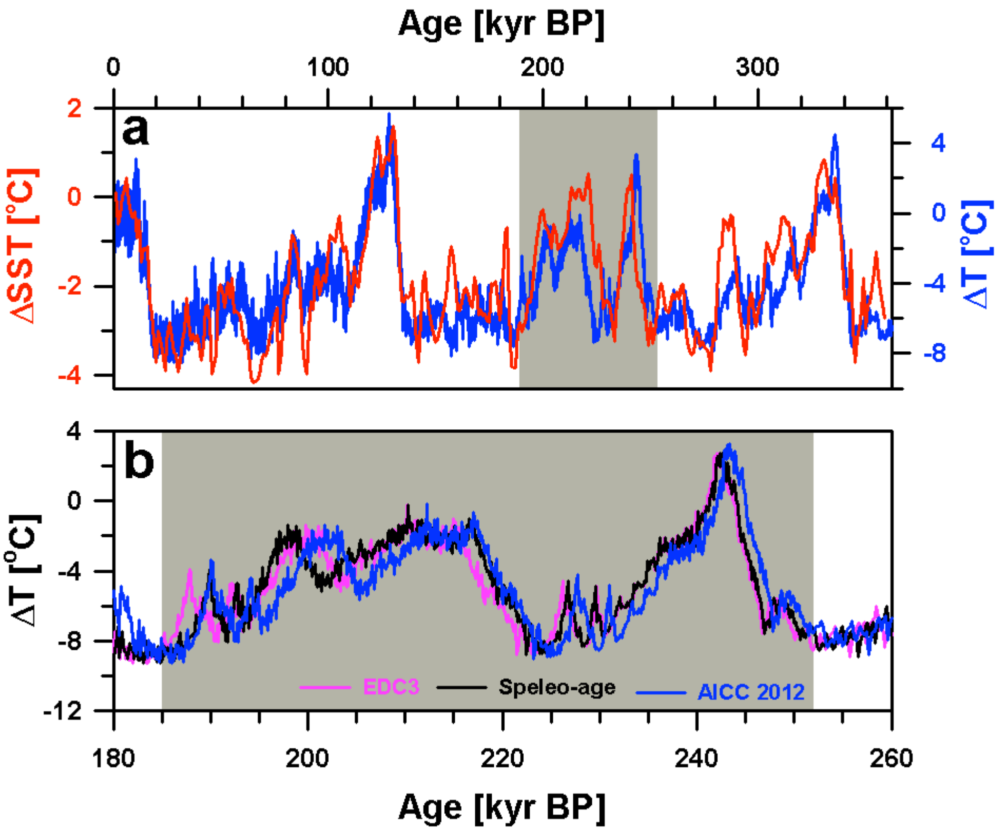

Supplementary Figure S6. Records of Solomon MD05-2925 ΔSST and Antarctic ΔT. (**a**) Comparison of MD05-2925 ΔSST (red) and Antarctic ΔT (blue) records over the past 360 kyr BP. Antarctic record is in AICC2012 time scale[7]. The gray window is marine isotope stage (MIS) 7 period, enlarged in (**b**). (**b**) Detailed MIS 7 Antarctic ΔT record with different age models of AICC2012 (refs. 10,11) (blue), Speleo-age[12] (black) and EDC3[13] (pink).

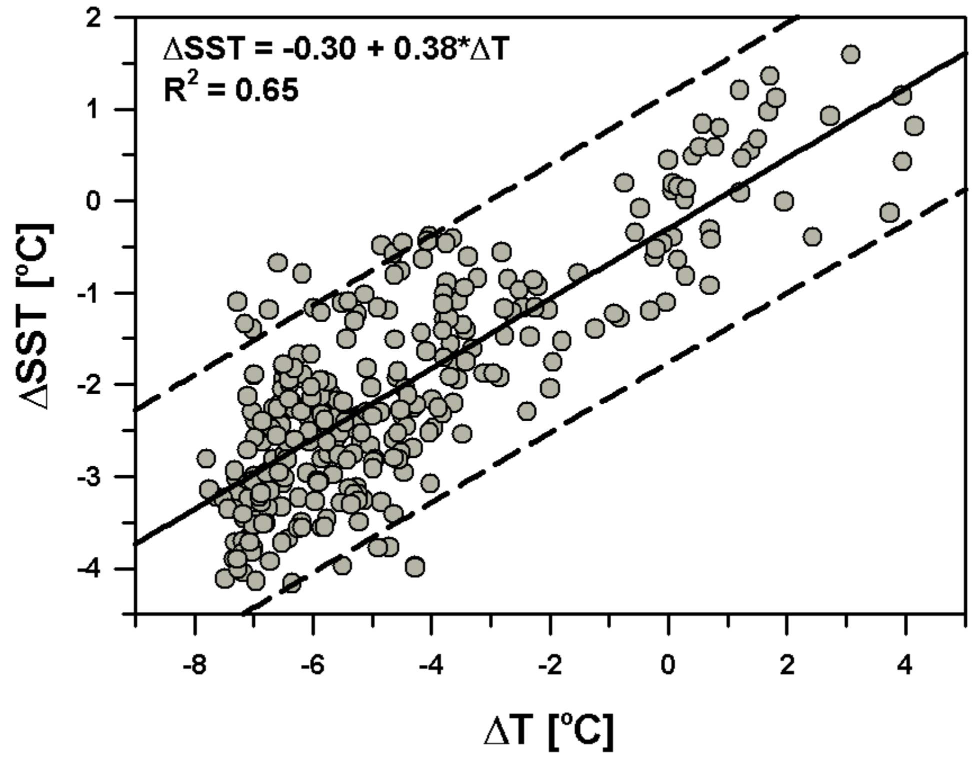

Supplementary Figure S7. A plot of Solomon MD05-2925 ΔSST versus Antarctic ΔT. The ΔSST and ΔT data were resampled into 1-kyr resolution, and the potential chronological problematic period during the MIS 7 (252-185 kyr BP, Supplementary Figure S6b) is omitted.

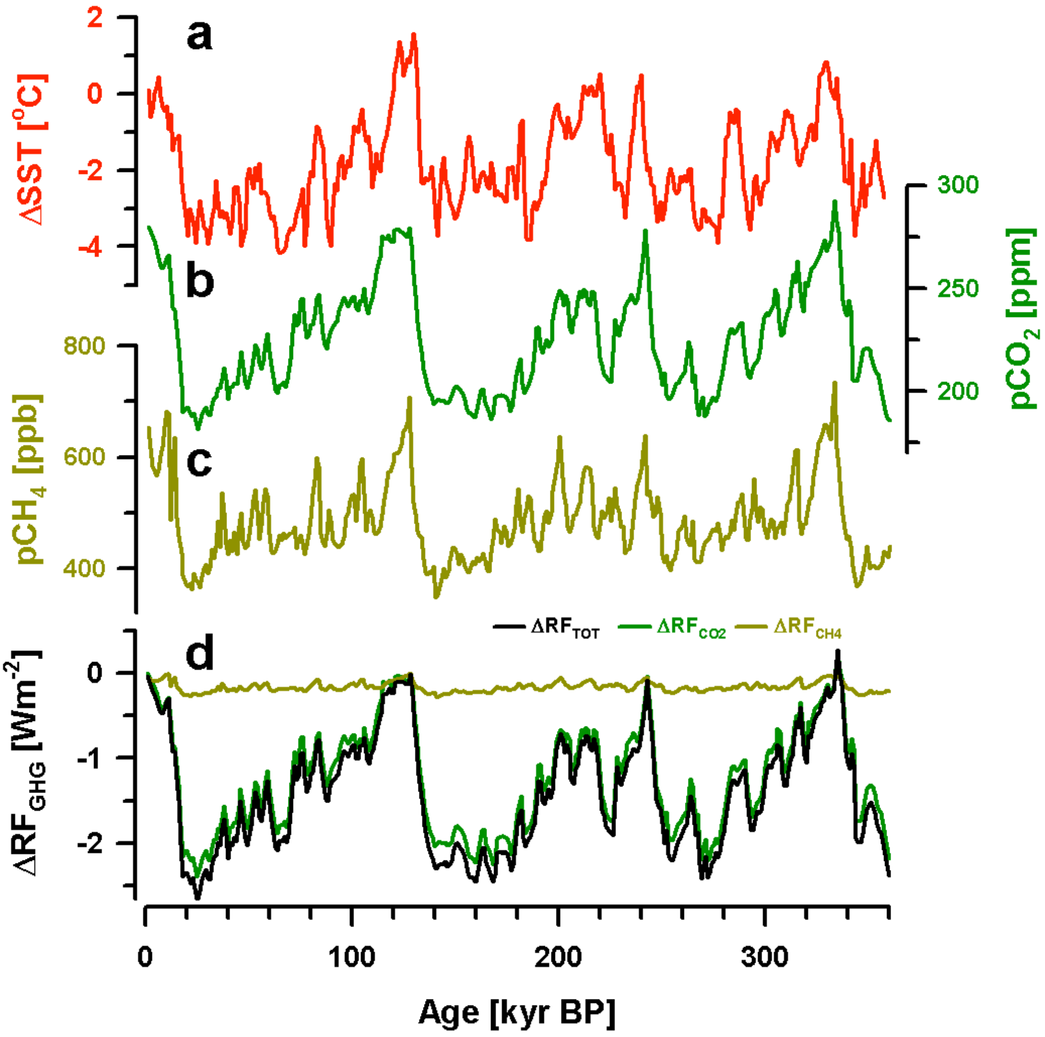

Supplementary Figure S8. Proxy-inferred records of MD05-2925 planktonic foraminiferal, *G. ruber*, and ice core. (**a**) Solomon Sea SST anomalies (ΔSST). (**b**) Atmospheric pCO_2 and (**c**) CH_4 (ref. 8). (**d**) Calculated ΔRF_{GHG}. Dark yellow and green lines in (**d**) represent the ΔRF_{GHG} sequences induced by atmospheric CO_2 and CH_4, respectively. Black line denotes the total ΔRF_{GHG}. All data are re-sampled into 1-kyr resolution.

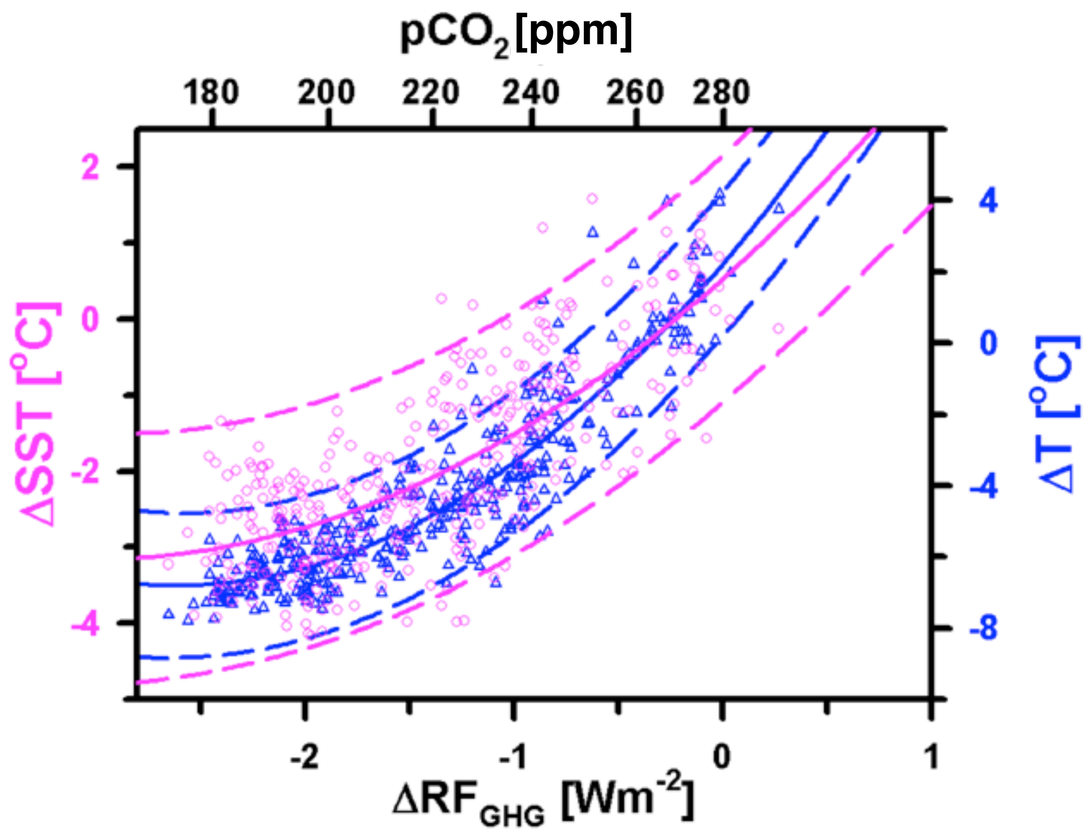

Supplementary Figure S9. Comparison between Antarctic temperature and Solomon thermal sensitivities. Blue triangles represent Antarctic ΔT (ref. 7) and pink circles are Solomon ΔSST data. Solid and dashed lines represent regression and 95% confidence lines, respectively.

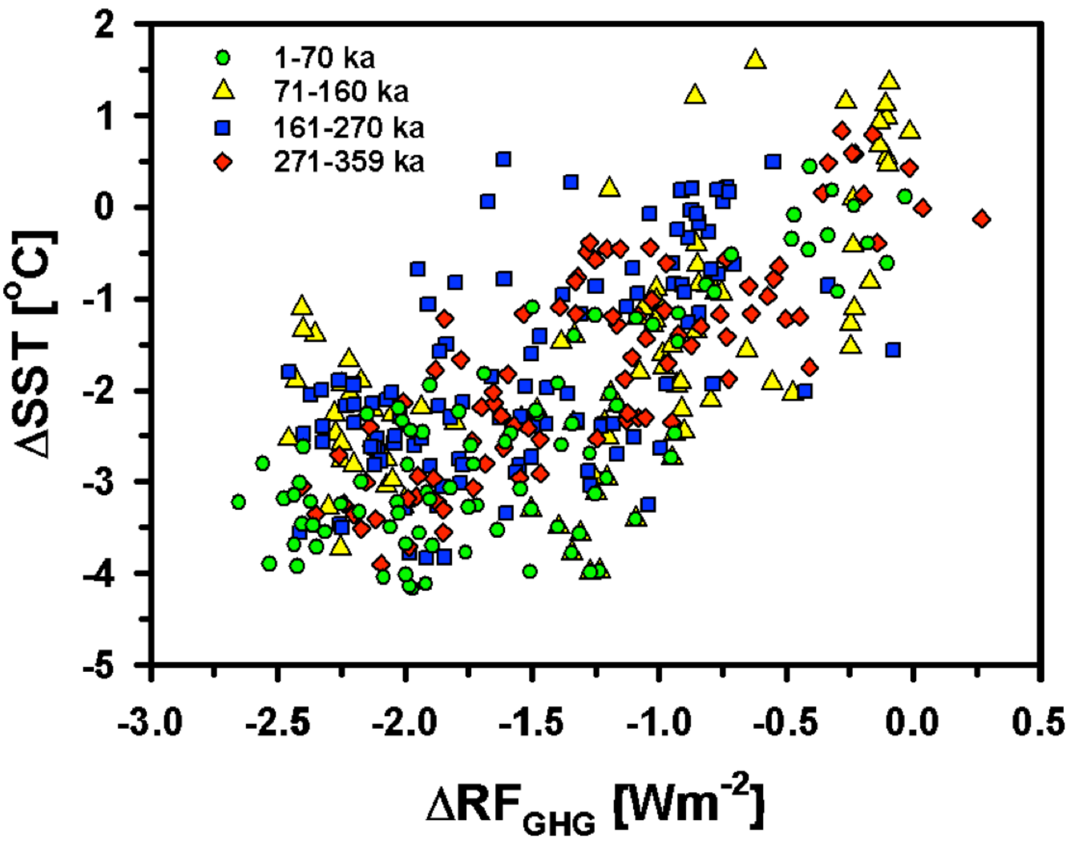

Supplementary Figure S10. A plot of Solomon ΔSST versus ΔRF_{GHG} during the past 360 kyr BP. Data are grouped by four G/IG cycles at 1-70 (green circles), 71-160 (yellow triangles), 161-270 (blue squares), and 271-360 (red diamonds) kyr BP.

Supplementary Table S1. Depth-age pairs of control points for core MD05-2925*.

Depth (cm)	Age (kyr BP)
**534**	58
**582**	75
**777**	112
**832**	130
**877**	135
**992**	167
**1122**	192
**1177**	205
**1205**	216
**1262**	232
**1432**	261
**1515**	283
**1570**	293
**1682**	317
**1738**	333
**1802**	341
**1842**	350

*Radiocarbon dates <30 kyr BP were reported[2].

Supplementary Table S2. Phase relationships among astronomical parameters, ice volume, SST, ΔT, CO_2, and CH_4.

	MD05-2925 SST					
	Precession			Obliquity		
Astronomical parameters[a]	-2.2 ± 0.7*			-3.3 ± 1.1		
LR04[b]	2.8 ± 1.1			4.2 ± 1.4		
	AICC2012[c]	Speleo-age[d]	EDC3[e]	AICC2012	Speleo-age	EDC3
ΔT[f]	-0.6 ± 1.1	1.3 ± 1.8	1.6 ± 0.9	0.4 ± 1.2	1.3 ± 1.5	2.5 ± 1.3
CO_2[g]	0.9 ± 1.1	3.5 ± 1.8	3.0 ± 1.2	2.5 ± 1.8	3.7 ± 2.6	4.7 ± 2.5
CH_4[h]	-1.1 ± 1.4	-0.9 ± 1.3	-0.2 ± 1.4	0.8 ± 1.8	0.5 ± 2.0	1.5 ± 1.6

*Negative values represent MD05-2925 SST lag, and positive ones represent MD05-2925 lead. The unit is thousand years (kyrs).

[a]*Laskar et al.*[14]
[b]*Lisiecki and Raymo*[4]
[c]*Bazin et al.*[10]; *Veres et al.*[11]
[d]*Barker et al.*[12]
[e]*Parrenin et al.*[13]
[f]*Jouzel et al.*[7]
[g]*Lüthl et al.* [8]
[h]*Loulergue et al.*[15]

Supplementary Table S3. Linear versus non-linear fits of sensitivities in each research sites. The unit of sensitivity is $^{o}C\ (W\ m^{-2})^{-1}$.

Sites	Linear fit	Non-linear fit	
		<220 ppm group	>220 ppm group
MD05-2925	1.44 ± 0.12*	0.62 ± 0.33	1.83 ± 0.17
ODP806	0.70 ± 0.10	0.53 ± 0.31	0.40 ± 0.26
MD97-2140	0.88 ± 0.21	-0.20 ± 0.15	1.01 ± 0.57
ODP871	0.65 ± 0.08	0.26 ± 0.19	0.94 ± 0.16
TR163-19	1.07 ± 0.17	0.51 ± 0.21	1.29 ± 0.50
ODP1240	1.45 ± 0.12	0.61 ± 0.14	2.35 ± 0.17

* All errors are in 1σ range.

Supplementary Table S4. The mean values of transition periods in the cluster analysis from each site. Note that ΔRF_{GHG} caused by pCO_2 from 210 to 230 ppm are -1.60 to -1.09 W m^{-2} (ref. 16).

Sites	Mean values of transition periods (W m^{-2})	Error (W m^{-2}, 1σ)
MD05-2925	-1.49	0.28
ODP806	-1.45	0.20
MD97-2140	-1.01	0.25
ODP871	-1.38	0.63
TR163-19	-1.20	0.14
ODP1240	-1.23	0.10

Supplementary References

1. Wessel, P., Smith, W. H. F., Scharroo, R., Luis, J. F. & Wobbe, F. Generic Mapping Tools: Improves versions released. *EOS Trans. AGU*, **94**, 40-410 (2013).
2. Lo, L. *et al.*, Millennial meridional dynamics of the Indo-Pacific Warm Pool during the last termination. *Clim. Past* **10**, 2253-2261 (2014).
3. Liu, Y, *et al.* Obliquity pacing of the western Pacific Intertropical Convergence Zone over the past 282,000 years. *Nature Communications* **6**, doi: 10.1038/ncomms10018 (2015).
4. Lisiecki, L. E. & Raymo, M. E. A Pliocene-Pleistocene stack of 57 globally distributed benthic $\delta^{18}O$ records. *Paleoceanography* **20**, PA1003 (2005).
5. Shackleton, N. J. & Opdyke, N. D. Oxygen isotope and palaeomagnetic stratigraphy of equatorial Pacific core V28-238: Oxygen isotope temperatures and ice volumes on a 10^5 year and 10^6 year Scale. *Quat. Res*. **3**, 39-55 (1973).
6. Oba, T. *et al.* Paleoceanographic change off central Japan since the last 144,000 years based on high-resolution oxygen and carbon isotope records. *Global Planet. Change* **53**, 5-20 (2006).
7. Jouzel, J. *et al*., Orbital and millennial Antarctic climate variability over the past 800,000 years. *Science* **317**, 793-796 (2007).
8. Lüthi, D. *et al.* High-resolution carbon dioxide concentration record 650,000–800,000 years before present. *Nature* **435**, 379-382 (2008).
9. Howell, P. ARAND time series and spectral analysis package for the Macintosh, Brown University. IGBP PAGES/World Data Center for Paleoclimatology Data Contribution Series #2001-031. NOAA/NGDC Paleoclimatology Program, Boulder, Colorado, USA. (2001).
10. Bazin, L. *et al.* An optimized multi-proxy, multi-site Antarctic ice and gas orbital chronology (AICC2012): 120-800 ka. *Clim. Past* **9**, 1715-1731 (2013).
11. Veres, D. *et al.* The Antarctic ice core chronology (AICC2012): An optimized multi-parameter and multi-site dating approach for the last 120 thousand years. *Clim. Past* **9**, 1733-1748 (2013).
12. Barker, S. *et al.* 800,000 Years of Abrupt Climate Variability. *Science* **334**, 347-351 (2011).
13. Parrenin, F. *et al.* The EDC3 chronology for the EPICA Dome C ice core. *Clim. Past* **3**, 485-497 (2007).
14. Laskar, J. *et al.* A long term numerical solution for the insolation quantities of the Earth. *Astron. Astrophys.* **428**, 261-285 (2004).
15. Loulergue, L. *et al.* Orbital and millennial-scale features of atmospheric CH_4 over the past 800,000 years. *Nature* **453**, 383-386 (2008).
16. Ramaswamy, V. *et al.* Radiative forcing of climate change in, *Climate Change 2001: The Scientific Basis*, Houghton, J. T. *et al.* eds., Cambridge University Press, 319-416 (2001).
